# A Manual of the Injuries and Surgical Diseases of the Mouth, Face and Jaws

**Published:** 1897-11

**Authors:** 


A MANUAL OF THE INJURIES AND SURGICAL DISEASES OF THE MOUTH, FACE AND JAWS. By John Sayre Marshall, M. D., Former Professor of Dental Pathology and Oral Surgery in the Dental Department of Northwestern University, etc. Publishers: The S. S. White Dental Manufacturing Co., Philadelphia. 1897.

The contents consist of Chapters consisting of "Surgical Bacteriology," "Inflammation," "Abscess," "Ulceration," "Necrosis, Caries and Gangrene," "Traumatic Inflammatory Fever," "Septicemia," "Pyemia," "Erysipelas," "Tetanus," "Shock and Collapse," "Ligatures, Sutures and Suturing," "Wounds and Their Treatment," "Gunshot Wounds," "Fractures of the Maxillæ," "Dislocations of the Inferior Maxillæ," "Anchylosis of the Jaws," "Necrosis of the Jaws," "Stomatitis," "Surgical Tuberculosis," Diseases of the Maxillary Sinus," "Cystic Tumors of the Maxillary Sinus," Diseases of the Salivary Glands," Neuralgia and Treatment of Trifacial Neuralgia," "Congenital Fissures of the Lip and Vault of the Mouth," "Tumors," "Tumors of the Face, Mouth and Jaws," and Retention Cysts; the whole forming a most excellent treatise on oral surgery; useful alike to the student and practitioner of both medicine and dentistry.

A long acquaintance with the author, led us to expect an admirable work on oral surgery when we first learned of his intention to prepare one, and our expectations have been more than realized. No one more competent to perform such a task than Dr. Marshall could have been selected, as his intellectual qualities and extensive hospital experience, have eminently fitted him to prepare and arrange the material of which this work is composed.

Its subjects are exhaustively as well as scientifically treated, and the work must be regarded as the best of this kind that has ever been published, and it will always remain as a standard treatise useful to all engaged in the practice of oral and general surgery.

The publishers deserve great credit for the excellent style in which the work appears, which consists of 716 pages of text illustrated by 364 engravings.

